# Role of biochemical markers as adjuncts to the Fagerström test in evaluating nicotine dependence: A retrospective observational study

**DOI:** 10.18332/tpc/216111

**Published:** 2026-07-16

**Authors:** Laura Gil-Pintor, Enrique Barrueco Otero, Vanesa Hidalgo-Sierra, Rosa Cordovilla Pérez, Miguel Ángel Hernández-Mezquita

**Affiliations:** 1 Department of Pulmonology, Salamanca University Hospital, Salamanca, Spain; 2 Ciudad Rodrigo Primary Care Health Center, Ciudad Rodrigo, Spain; 3 Elena Ginel Díez Primary Care Health Center (Tejares), Salamanca, Spain

**Keywords:** tobacco cessation, Fagerström test for nicotine dependence (FTND), cotinine, CO, COHb

## Abstract

**INTRODUCTION:**

Nicotine dependence assessment is essential in smoking cessation programs. The Fagerström test for nicotine dependence (FTND) is widely used, but its subjective nature limits its accuracy. Biomarkers, such as cotinine, exhaled carbon monoxide (CO), and carboxyhemoglobin (COHb), provide objective measures of tobacco exposure and complement information for disease management. This study aimed to evaluate the association between biochemical markers and nicotine dependence measured by FTND in a large population of smokers attending a smoking cessation program, and to determine which biomarkers best reflect dependence levels.

**METHODS:**

This retrospective observational study was conducted in the Health Area of Salamanca, including the Smoking Cessation Unit of Salamanca University Hospital and all Primary Care centers in the metropolitan area, from January 2009 to July 2021. A total of 3820 smokers were included. Demographic data, smoking history, and nicotine dependence levels were recorded in patients’ medical history at the first visit to the Smoking Cessation Unit. Biomarkers, including serum cotinine, exhaled CO, and COHb, were measured, and the association between these biomarkers and baseline FTND scores was analyzed. Although patients were followed for at least six months as part of a broader smoking cessation program, follow-up data were not included in the analysis.

**RESULTS:**

Cotinine levels showed the strongest correlation with FTND (r=0.302, p<0.001), followed by COHb (r=0.234, p<0.001) and CO (r=0.219, p<0.001). When stratified by dependence level, mean concentrations of cotinine, CO, and COHb increased progressively from low to high dependence categories, with statistically significant differences across all groups (p<0.001).

**CONCLUSIONS:**

Biomarkers showed consistent associations with nicotine dependence measured by the FTND, with cotinine demonstrating the strongest association. Further studies are needed to confirm these findings and to provide sufficient evidence on the utility of biochemical markers in assessing nicotine dependence.

## INTRODUCTION

Smoking remains among the primary preventable causes of morbidity and mortality worldwide, accounting for over 8 million deaths annually, according to the World Health Organization (WHO)^1^. The prevention, diagnosis, and management of tobacco use constitute a multidisciplinary activity shared between Primary Care and other medical specialties. Our working group, composed of family physicians and pulmonologists, has established extensive experience in clinical practice and research collaboration in this field^2^.

Tobacco causes numerous diseases and leads to physical and psychological dependence, which makes it difficult to quit^1^. Physical nicotine dependence is commonly assessed using the Fagerström test for nicotine dependence (FTND). The FTND consists of six questions and classifies dependence as: mild (0–4 points), moderate (5–6 points), or severe (≥ 7 points)^2^. Its psychometric properties have been questioned in recent studies, and the subjective nature of the test may limit its clinical utility, as nicotine dependence is a complex process that includes both physical and psychological aspects that are difficult to quantify^3^. The combined use of this test (FTND) with objective parameters, such as biochemical markers, could help overcome these limitations.

In this context, biological markers such as exhaled carbon monoxide (CO), carboxyhemoglobin (COHb), and cotinine may serve as objective tools for quantifying tobacco exposure and assessing nicotine dependence^4^.

CO is a colorless and odorless gas produced by tobacco combustion^5-9^. Its measurement in exhaled air enables the estimation of recent exposure to tobacco smoke, as there is a direct relationship between the number of cigarettes smoked and CO levels in exhaled air^10^. Regular smokers typically exhibit CO concentrations of 8–10 ppm or higher, with this value serving as the commonly used cutoff point. The sensitivity and specificity of this measure are approximately 90%, although the cutoff point may vary depending on environmental factors^8,9^.

Occasional smokers generally present CO levels below 10 ppm but consistently above six ppm, whereas non-smokers rarely present levels exceeding 6 ppm^4^. Most of the CO binds reversibly to hemoglobin (Hb), forming carboxyhemoglobin (COHb). The affinity of CO for hemoglobin is 200 to 250 times greater than that of oxygen; however, COHb is fully dissociable, and CO is released and eliminated through the lungs once exposure ceases^10-13^.

Cotinine is the primary metabolite of nicotine and has a half-life of 11–37 hours, compared to 2–5 hours for CO, making it a more reliable indicator of tobacco consumption^4^. Its sensitivity and specificity for distinguishing smokers from non-smokers are high, as well as for quantifying consumption: regular smokers typically exhibit serum levels between 200 and 400 ng/mL, light smokers present levels between 40 and 50 ng/mL, and non-smokers have levels below 10 ng/mL^14-16^.

In smokers, cotinine levels correlate with the number of cigarettes smoked per day, smoking history, and CO levels. Although this statement by Benowitz^17^ dates back to the 1980s, it has been validated by various authors in subsequent years.

Regarding biochemical markers, it is important to note that for a smoker to develop physical nicotine dependence, nicotine must be chronically present in the bloodstream. Since the FTND is designed to assess the degree of physical nicotine dependence, its scores are expected to positively correlate with blood levels of cotinine or other biochemical markers of tobacco consumption^18^.

Studies examining the relationship between exhaled CO concentration and FTND scores typically rely on a single measurement^19,20^. However, as Pérez Trullen et al.^21^ point out, a single, isolated CO reading is not a reliable indicator of chronic nicotine consumption. Due to its short half-life (2–5 hours), CO levels are significantly influenced by the time elapsed since the last cigarette was smoked^18,22^. For this reason, the longer half-life of cotinine (15–20 hours) makes its blood concentration less sensitive to the timing of the last cigarette compared to CO measurement in exhaled air. Consequently, cotinine is presumably a more appropriate biochemical marker for chronic nicotine intake and, therefore, a more suitable parameter for correlating with dependence.

The use of biochemical markers, in combination with dependence assessment tools, may improve the design and effectiveness of personalized treatments and enable more precise patient follow-up. This study evaluates the usefulness of these biochemical markers as a complement to the FTND in smokers initiating smoking cessation programs.

## METHODS

### Study design and population

A retrospective observational study was conducted in the Health Area of Salamanca, including the Smoking Cessation Unit of the University Hospital of Salamanca and all 36 primary care health centers in the metropolitan area and province, covering urban, semi-urban, and rural populations. Patients were included between January 2009 and July 2021 under standardized clinical protocols developed jointly by the Smoking Cessation Unit and primary care teams.

Patients were consecutively recruited among those referred from Primary Care centers who were smokers aged ≥18 years and expressed an intention to quit smoking. The retrospective design was chosen to allow comprehensive analysis of long-term patterns and biochemical marker data already collected in clinical practice. A total of 3820 smokers met the inclusion criteria and were included in the analysis.

This retrospective study was conducted in accordance with the Declaration of Helsinki. It includes routinely collected clinic data from the Smoking Cessation Unit database at the University Hospital of Salamanca, without the performance of any additional tests. The biological samples used in the study had been collected as part of standard clinical care at our hospital.

The study was approved by the Ethics Committee of the University Hospital of Salamanca (approval code PI20210 827).

### Characteristics of participants

Demographic variables, including sex (male, female) and age (years), tobacco consumption variables, including cigarettes per day, years of smoking, and pack-year index (PYI), biochemical variables, including serum cotinine (ng/mL), exhaled carbon monoxide (CO) (ppm), and carboxyhemoglobin (COHb) (%), and nicotine dependence level assessed by the Fagerström test for nicotine dependence (FTND, score 0–10) were collected from the anonymized database of the Smoking Cessation Unit. Smoking history and FTND scores were self-reported by patients using standardized questionnaires during their first visit.

Sex was determined based on biological characteristics, and gender information was not recorded. Race and ethnicity were not collected because the study population was drawn from a single, well-defined community, rendering these variables irrelevant to the study objectives. Only sex was considered as a potential confounder in the analyses.

### Data collection and measurements

All variables were collected according to standardized clinical protocols routinely used in daily practice at the Smoking Cessation Unit and affiliated Primary Care centers. These protocols included detailed patient history, structured assessment of smoking habits, measurement of biochemical markers under controlled conditions, and standardized administration of the FTND questionnaire. Biochemical markers were measured using validated methods: serum cotinine levels were determined in blood samples using a commercial Enzyme-Linked Immunosorbent Assay (ELISA) kit, exhaled CO was measured with calibrated breath analyzers, and COHb concentrations were obtained through routine laboratory procedures. Nicotine dependence was evaluated with the Fagerström test for nicotine dependence (FTND), administered by trained staff according to established guidelines. All procedures followed standard operating procedures to ensure consistency and reproducibility, and were in line with the 2019 Society for Research on Nicotine and Tobacco (SRNT) guidelines for biochemical verification of tobacco use and abstinence23.

### Statistical analysis

Bivariate analyses (Pearson correlation) were conducted to examine relationships between biochemical markers and other variables. Subsequently, associations between biochemical marker levels and nicotine dependence (categorized as high, moderate, or low based on FTND scores) were evaluated using analysis of variance (ANOVA) with multiple comparisons. Bonferroni correction and Dunnett’s t-test were applied to control for Type I error. A p<0.001 was considered statistically significant.

All analyses were conducted using IBM SPSS Statistics, version 25.0. Statistical methods and software were chosen to ensure that other researchers with access to the data could reproduce the results.

## RESULTS

### Study population

Of the 3820 patients included, 2025 (53%) were men and 1795 (47%) women, with a mean age of 48.6 ± 11.4 years. The average tobacco consumption was 24.5 ± 11.5 cigarettes per day, with a mean pack-years index (PYI) of 40.1 ± 24.1 and a mean duration of previous smoking history of 31.6 ± 11.6 years.

### Nicotine dependence

As shown in Table 1, the mean FTND score in the study population was 6.68 ± 1.91. According to the FTND scale, 56% of patients were classified as having high dependence, 31.4% as moderate dependence, and 12.6% as low dependence.

**Table 1:** Biochemical marker levels and nicotine dependence averages of sample, Salamanca Spain, January 2009–July 2021 (N=3820)

	*Global mean values*	*Mean values by dependence levels*	*Dependence levels (FTND)*
**Cotinine** (ng/mL)	365.11 ± 175.37	262.170 ± 154.83	Low
		336.11 ± 164.31	Moderate
		402.767 ± 173.20	High
**CO** (ppm)	20.50 ± 13.93	14.10 ± 11.27	Low
		18.83 ± 13.20	Moderate
		22.49 ± 14.64	High
**COHb** (%)	3.81 ± 2.05	2.78 ± 1.76	Low
		3.55 ± 1.97	Moderate
		4.12 ± 2.13	High
**Fagerström test**	6.68 ± 1.91		

CO: carbon monoxide. COHb: carboxyhemoglobin. FTND: Fagerström test for nicotine dependence. Values are given as mean with standard deviation.

### Biochemical markers

The mean blood cotinine level was 365.1 ± 175.3 ng/mL. Stratified by dependence level according to the FTND, mean cotinine concentration was 402.78 ± 173.20 ng/mL in those with high dependence, 336.11 ± 164.31 ng/mL in those with moderate dependence, and 262.17 ± 154.83 ng/mL in patients with low dependence. Differences among the three groups were statistically significant (p<0.001).

The mean level of exhaled carbon monoxide (CO) was 20.5 ± 13.9 ppm, with values of 22.4 ± 14.6 ppm for high dependence, 18.8 ± 13.2 ppm for moderate dependence, and 14.1 ± 11.3 ppm for low dependence (p<0.001).

The mean carboxyhemoglobin (COHb) concentration was 3.8 ± 2%, with 4.1 ± 2.1% for high dependence group, 3.5 ± 1.9% in the moderate dependence group, and 2.7 ± 1.7% for low dependence. The differences among the three groups were statistically significant (p<0.001).

### Correlations and associations

**Figure 1:**
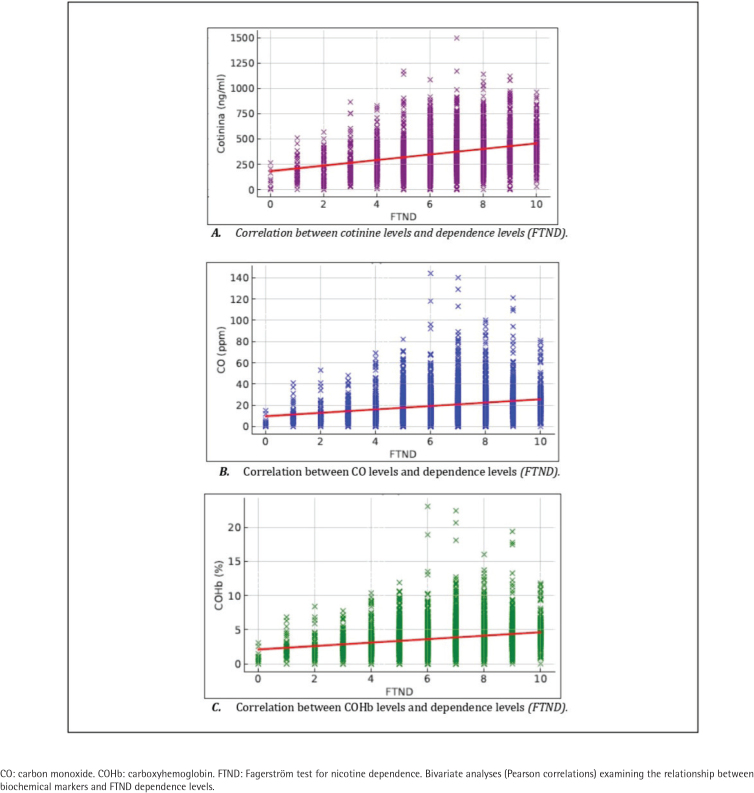
Correlation between biochemical levels and nicotine dependence (FTND), Salamanca, Spain, January 2009–July 2021 (N=3820)

**Figure 2:**
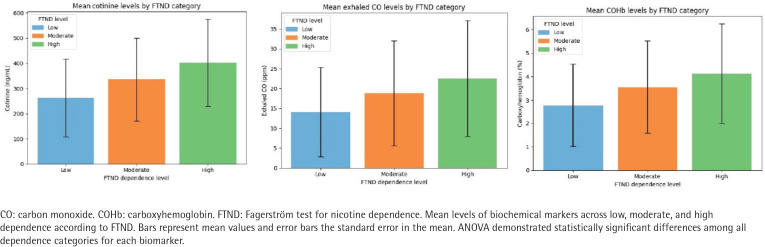
Evolution of biochemical markere levels based on nicotine dependence according to the FTND, Salamanca, Spain, January 2009–July 2021 (N=3820)

**Figure 3:**
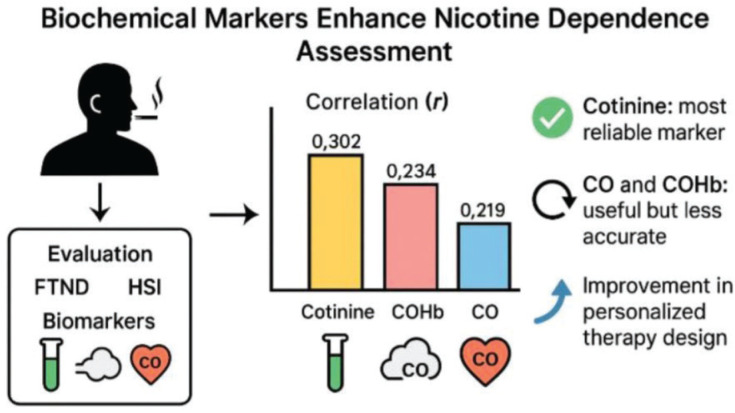
Role of biochemical marker as adjuncts to the Fagerström test in evaluating nicotine dependence, Salamanca, Spain, January 2009–July 2021 (N=3820)

Cotinine showed a significant correlation with FTND scores (r=0.302, p<0.001), indicating a consistent association with chronic nicotine exposure (Figure 1A). CO and COHb showed moderate correlations with FTND (r=0.219, p<0.001 and r=0.234, p<0.001, respectively) (Figures 1B and 1C, and Table 2). When analyzed individually using analysis of variance (ANOVA), the relationship between biochemical marker levels and tobacco dependence levels (high, moderate, or low) showed statistically significant differences across all categories (p<0.001). These results are summarized in Table 3 and Figure 2. The role of biomarkers as adjuncts to the FTND in evaluating nicotine dependence is displayed in Figure 3.

**Table 2:** Pearson correlation between biochemical markers and dependence according to FTND, Salamanca, Spain, January 2009–July 2021 (N=3820)

		*Cotinine*	*FTND*	*CO*	*COHb*
**Cotinine** (ng/mL)	Pearson correlation (r)	1	0.302	0.333	0.345
	p		<0.001	<0.001	<0.001
	N		3563	3482	3482
**CO** (ppm)	Pearson correlation (r)	0.333	0.219	1	0.996
	p	<0.001	<0.001		<0.001
	N	3482	3648		3655
**COHb** (%)	Pearson correlation (r)	0.345	0.234	0.996	1
	p	<0.001	<0.001	<0.001	
	N	3482	3648	3655	

CO: carbon monoxide. COHb: carboxyhemoglobin. FTND: Fagerström test for nicotine dependence.

**Table 3:** Statistical significance of the relationship between biochemical markers and dependence levels according to the FTND scale, Salamanca, Spain, January 2009–July 2021 (N=3820)

*Multiple comparisons*								
				*Difference of means (I-J)*	*SE*	*p*	*95% CI*	
							*Lower*	*Upper*
**Cotinine** (ng/mL)	Bonferroni	Low	Moderate	-73.94	9.51	<0.001	-96.72	-51.15
			High	-140.60	8.92	<0.001	-161.96	-119.24
		Moderate	Low	73.94	9.51	<0.001	51.15	96.72
			High	-66.66	6.27	<0.001	-81.67	-51.65
		High	Low	140.60	8.92	<0.001	119.24	161.96
			Moderate	66.66	6.27	<0.001	51.65	81.67
	Dunnett’s t-test (bilateral)	Moderate	Low	73.94	9.51	<0.001	53.36	94.51
		High	Low	140.60	8.92	<0.001	121.31	159.88
	Dunnett’s t-test (bilateral)	Moderate	Low	4.40	1.27	<0.001	1.66	7.14
		High	Low	18.59	1.19	<0.001	16.03	21.16
**CO (ppm)**	Bonferroni	Low	Moderate	-4.72	0.77	<0.001	-6.56	-2.88
			High	-8.39	0.72	<0.001	-10.12	-6.66
		Moderate	Low	4.72	0.77	<0.001	2.88	6.56
			High	-3.67	0.51	<0.001	-4.89	-2.45
		High	Low	8.39	0.72	<0.001	6.66	10.12
			Moderate	3.67	0.51	<0.001	2.45	4.89
	Dunnett’s t-test (bilateral)	Moderate	Low	4.72	0.77	<0.001	3.06	6.39
		High	Low	8.39	0.72	<0.001	6.83	9.95
**COHb (%)**	Bonferroni	Low	Moderate	-0.77	0.11	<0.001	-1.05	-0.50
			High	-1.34	0.11	<0.001	-1.59	-1.08
		Moderate	Low	0.77	0.11	<0.001	0.50	1.05
			High	-0.56	0.08	<0.001	-0.75	-0.39
		High	Low	1.34	0.11	<0.001	1.08	1.59
			Moderate	0.57	0.08	<0.001	0.39	0.75
	Dunnett’s t-test (bilateral)	Moderate	Low	0.77	0.1135	<0.001	0.528	1.019
		High	Low	1.33	0.1063	<0.001	1.108	1.568

CO: carbon monoxide. COHb: carboxyhemoglobin. FTND: Fagerström test for nicotine dependence. SE: standard error. Associations between biochemical marker levels and nicotine dependence (categorized as high, moderate, or low based on FTND scores) were evaluated using analysis of variance (ANOVA) with multiple comparisons. Bonferroni correction and Dunnett’s t-test were applied to control for Type I error. A p<0.05 was considered statistically significant.

## DISCUSSION

In our study, the relationship between CO and COHb levels and the FTND showed a positive association (r=0.219 for CO and r=0.234 for COHb). Similarly, Clemente et al.22 reported significant correlations between CO and FTND (r=0.5853; p=0.0001), and between COHb and FTND (r=0.549; p=0.0001). These findings indicate that higher levels of CO and/or COHb are associated with higher FTND scores, which is consistent with the existing literature. However, it should be noted that the correlation values observed in our study are relatively low, although statistically significant.

Published studies on the relationship between tobacco consumption biomarkers and nicotine dependence levels are scarce, highlighting the importance of using biochemical markers that accurately reflect the degree of chronic nicotine exposure^23,24^. Since the FTND is designed to measure the degree of physical nicotine dependence, its scores are expected to correlate positively with biochemical marker levels and can therefore be considered not only direct indicators of consumption but also indirect indicators of dependence that may complement the information provided by the FTND.

The relationship between CO and dependence established by the FTND shows significant variation in the literature, with correlation coefficients ranging from 0.23 to 0.88, which does not happen in the case of cotinine^25-27^. Benowitz et al.^28^ note that these differences are due to using single CO measurements to assess the association, so CO cannot be considered a good index of tobacco consumption. Although CO has an accumulated value throughout the day based on consumption, it has a very short half-life from the last cigarette, so its utility is limited^28,29^.

For this reason, cotinine has emerged as a more reliable indicator of tobacco consumption due to its longer half-life compared to CO and COHb^14^. However, the available literature on the relationship between tobacco dependence and cotinine levels is both limited and lacks recent evidence^18^. Despite this, the published findings are consistent with those of our study, showing significant and moderately strong associations between cotinine levels and the degree of dependence, as measured by the FTND. Pomerleau et al.^18^ reported a correlation of 0.42 (p<0.005) between cotinine levels and FTND scores. In our study, the correlation for cotinine on the FTND scale was 0.302, slightly lower than that reported by Pomerleau et al.^18^. In any case, the association observed with cotinine was stronger than that observed with CO and COHb.

The results of this study support the use of biochemical markers as complementary tools, rather than substitutes, for traditional nicotine dependence tests. Although the FTND is widely used, its subjective nature limits its accuracy, necessitating the complementation or integration of the information obtained with additional parameters. Cotinine shows a stronger correlation with nicotine dependence – as measured by the FTND – than exhaled CO or COHb, positioning it as a useful biochemical marker for quantifying physical nicotine dependence.

Nonetheless, the determination of CO and COHb involves less invasive procedures and is simpler, cheaper, and faster than cotinine measurement. Therefore, a cost-benefit analysis should be performed in each case to determine which test is most appropriate.

### Limitations

Several limitations should be considered when interpreting the results of this study. First, the retrospective observational design may be subject to biases in data collection and in completeness of records. Second, the study population consisted exclusively of patients referred from primary care centers who were motivated to quit smoking, which may limit the generalizability of the findings to all smokers. Third, although biochemical markers provide objective measures of tobacco exposure, factors such as inter-individual metabolic variability or recent exposure may have influenced the results. Finally, the study relied on routinely collected clinical data, which may not capture all potential confounders or behavioral factors influencing nicotine dependence. Despite these limitations, the large sample size and standardized protocols strengthen the reliability of the findings.

## CONCLUSIONS

The findings of this study highlight the potential of biochemical markers – particularly cotinine – as objective tools to complement traditional assessments of nicotine dependence, such as the FTND. Given cotinine’s stronger association with dependence levels, its integration into smoking cessation programs may enhance personalized treatment strategies. However, the practicality and lower cost of CO and COHb measurements make them valuable alternatives in resource-limited settings. Future research should explore cost-effectiveness analyses and long-term clinical outcomes to optimize the use of these biomarkers in routine smoking cessation interventions.

## Data Availability

The data supporting this research cannot be made available for privacy or other reasons.
